# Is double-crossed retrograde elastic stable intramedullary nailing an alternative method for the treatment of diaphyseal fractures in the adult humerus?

**DOI:** 10.1186/s10195-022-00662-7

**Published:** 2022-08-17

**Authors:** Hsuan-Hsiao Ma, Chao-Ching Chiang, Yu-Ping Su, Kun-Hui Chen

**Affiliations:** 1 Department of Orthopaedics and Traumatology, Taipei Veterans General Hospital, 201, Sec 2, Shih-Pai Road Taipei 112, Taipei, Taiwan; 2 Division of Orthopaedics, Department of Surgery, Taipei Veterans General Hospital Taitung Branch, Taitung, Taiwan; 3 Department of Orthopaedics, School of Medicine, National Yang Ming Chiao Tung University, Taipei, Taiwan; 4 Department of Orthopaedics, Taipei Veterans General Hospital Yuli Branch, Hualien, Taiwan; 5 Institute of Clinical Medicine, National Yang Ming Chiao Tung University, Taipei, Taiwan

**Keywords:** Adult diaphyseal humerus fracture, Closed reduction, Limited-contact dynamic compression plate, Open reduction, Titanium elastic nail

## Abstract

**Background:**

The aim of this study was to compare two techniques for the surgical treatment of diaphyseal fractures in the adult humerus: double-crossed retrograde elastic stable intramedullary nailing (DCR-ESIN) and limited-contact dynamic compression plate (LC-DCP).

**Methods:**

This was a retrospective study conducted at a single hospital. We included 122 patients with diaphyseal fractures of the humerus who had received DCR-ESIN or LC-DCP from January 2011 to January 2017. We compared union rates, union times, disabilities of the arm, shoulder, and hand (DASH) scores at the postoperative 1-year follow-up, and complications between the two groups.

**Results:**

Plating management was performed in 63 patients, while DCR-ESIN was performed in 59 patients. The union rate was higher in the DCR-ESIN group than in the LC-DCP group (100% vs. 90.5%; *p* = 0.052). The union time was shorter in the DCR-ESIN group than in the LC-DCP group (12.0 weeks vs. 14.8 weeks; *p* < 0.001). The intraoperative blood loss and operative time were less in the DCR-ESIN group than in the LC-DCP group (76.4 min vs. 129.5 min; *p* < 0.001; 60.9 ml vs. 244.8 ml; *p* < 0.001, respectively). The DCR-ESIN had superior results for the rate of overall complications (*p* = 0.006). At the 1-year follow-up, the DCR-ESIN group had better DASH scores than the LC-DCP group (*p* = 0.014).

**Conclusions:**

The DCR-ESIN technique, used to treat diaphyseal fractures of the humerus, has shorter operative times, less intra-operative blood loss, shorter union times, and better functional outcomes at 1-year follow-up than the LC-DCP technique. DCR-ESIN may be an alternative method for the surgical treatment of diaphyseal humeral fractures in adults.

## Introduction

While there are several methods of operative intervention for humeral diaphyseal fractures, the internal fixation methods can be broadly grouped into plating or intramedullary techniques. Recent studies introduced the interlocking nail technique, which is considered an effective method for humeral fixation; however, it can have some complications, such as shoulder movement restrictions due to the antegrade interlocking nails or because of impingement due to the proximal migration of nails, rotator cuff violations, adhesive capsulitis, or from unexplained causes [1–3]. These problems can be minimized by using retrograde techniques, but this also increases the risk of fractures at the olecranon fossa insertion point [4, 5]. As a result, limited-contact dynamic compression plates or locking compression plates are still the standard fixation methods for diaphyseal fractures of the adult humerus [6]. While elastic stable intramedullary nailing (ESIN) with titanium elastic nails (TENs) has been established as the primary method to surgically stabilize pediatric long-bone fractures [7], it has seldom been used to treat adult traumas.

Therefore, the aim of this study was to compare the outcomes of the double-crossed retrograde elastic stable intramedullary nailing (DCR-ESIN) technique with reduction and cerclage wire positioning if necessary with the outcomes of the limited-contact dynamic compression plate (LC-DCP) technique to treat diaphyseal fractures of the adult humerus. Our hypothesis was that the DCR-ESIN group would show comparable surgical outcomes to the LC-DCP group.

## Patients and methods

### Patients

This was a retrospective study conducted at a single hospital, and the protocol of this study was approved by the institutional review board (2019-04-003B). All the patients who presented with humeral shaft fractures and received surgery from January 2011 to January 2017 were included in this retrospective analysis. The inclusion criteria for patients in this study were humeral shaft fractures that required operative interventions and were treated with retrograde titanium elastic nails or those treated with LC-DCPs. Patients with intra-articular extensions of humeral shaft fractures, metaphyseal extensions to the proximal or distal humerus, pathological fractures, pediatric fractures (patients less than 18 years of age), periprosthetic fractures, and previous humeral surgeries or deformities were excluded. Fracture images were reviewed by the senior author, who has been well trained as an upper-limb orthopedic surgeon. The fractures were classified according to the Müller AO classification system and by location (proximal, middle, and distal third). Demographic and comorbidity data were collected from the medical records. A body mass index greater than 30 kg/m^2^ was considered to indicate obesity. The patients were assigned to the surgeon on call on the day they were delivered to our hospital.

### Surgical technique

A 4.5-mm LC-DCP was used in the plating group depending on the width of the bone, following appropriate AO principles. The choice of surgical approach—posterior or anterolateral—in the plating group was left at the discretion of the operating surgeon. In both approaches, the radial nerve was released and mobilized as needed to access the bone.

**Fig. 1:**
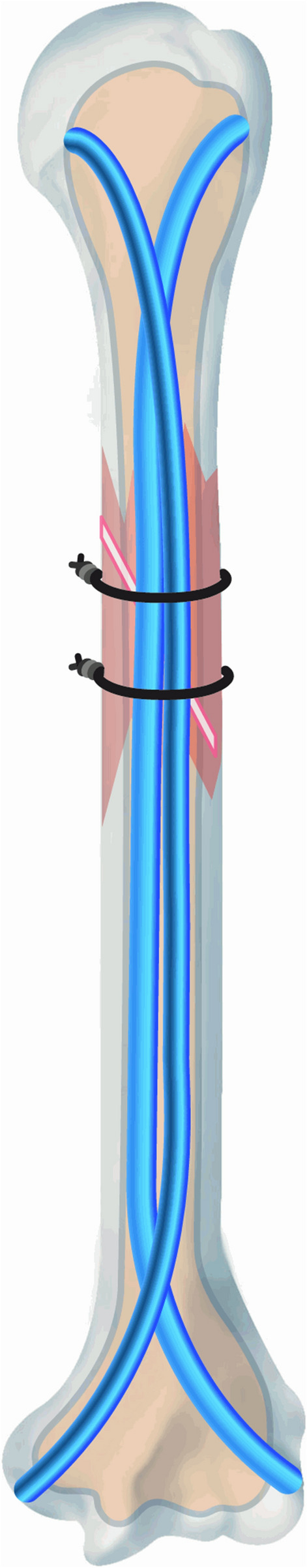
Illustration of the double-crossed retrograde elastic stable intramedullary nailing (DCR-ESIN) technique

The TENs used in the ESIN technique were inserted using the retrograde method. A small incision (0.5–1 cm) was used to find the bony structures of the medial and lateral epicondyles. The entry points were prepared using an awl or drill. The TENs, which had a diameter of 3.5 mm or 4.5 mm and were pre-bent to ensure maximum curvature, were localized at the fracture site and inserted. The lateral-side TEN was inserted first, followed by the medial-side TEN. The sum of the diameters of the two inserted TENs must be at least 70% of the diameter of the canal. Under C-arm control, a closed reduction was performed when the TENs reached the fracture site. Additional cerclage wire was applied through a mini-open incision without an open fracture site to remove the fracture hematoma when an acceptable reduction could not be achieved. The two TENs were advanced to within 1–2 cm of the proximal metaphysis to allow for good axial rotation control. Finally, the TENs were cut, impacted, and the ends of the TENs were trimmed to 0.5 cm from the bone to prevent skin irritation or ulnar nerve impingement (Fig. 1).

### Rehabilitation protocol

All the patients in both groups were instructed to keep their arms in a sling or a sugar-tong splint for 2 weeks. After 2 weeks, the patients were advised to remove the sling or sugar-tong splint 2–5 times per day for shoulder and elbow passive range of motion. From the fourth week onwards, they were encouraged to progress from passive-assisted to active exercises. All patients were prohibited from lifting weight or putting additional stresses on the affected limb for the first 3 months. Patients were discharged and were followed up at 4- to 6-week intervals until fracture union. In addition, patients were asked to complete the DASH questionnaire 1 year after surgery.

### Image evaluation

Unions were defined as cortical bridging at three of four cortices on orthogonal radiographs with the disappearance of fracture lines, the absence of pain at the fracture site, and with a return to full activities as well as the assessment from Westrick et al. [8]. Union was considered to have been achieved if the aforementioned criteria were fulfilled before 26 weeks. Delayed union was defined as union occurring after 26 weeks [9]. In addition, the angulated degree was measured by the senior author using anteroposterior (AP) and lateral views at the last follow-up. All follow-ups were performed at our institution with computer-archived radiographs in the UltraQuery system (Taiwan Electronic Data Processing, Sindian City, Taiwan).

### Outcome measurement

The primary outcome measures were fracture union, which was detected using follow-up radiographs, and complications after surgery, such as radial nerve injuries, wound infections, and implant-related complications, including backout and implant loosening or breakage. The secondary outcomes included 1-year DASH scores and postoperative angulation seen on radiographs.

### Statistics

All the statistical analyses were performed using SPSS version 24.0, and a two-sided probability of  < 0.05 was considered statistically significant. Differences between two groups were analyzed using independent paired *t*-tests for continuous variables and Fisher’s exact test and the chi-squared test for categorical variables.

## Results

**Fig. 2:**
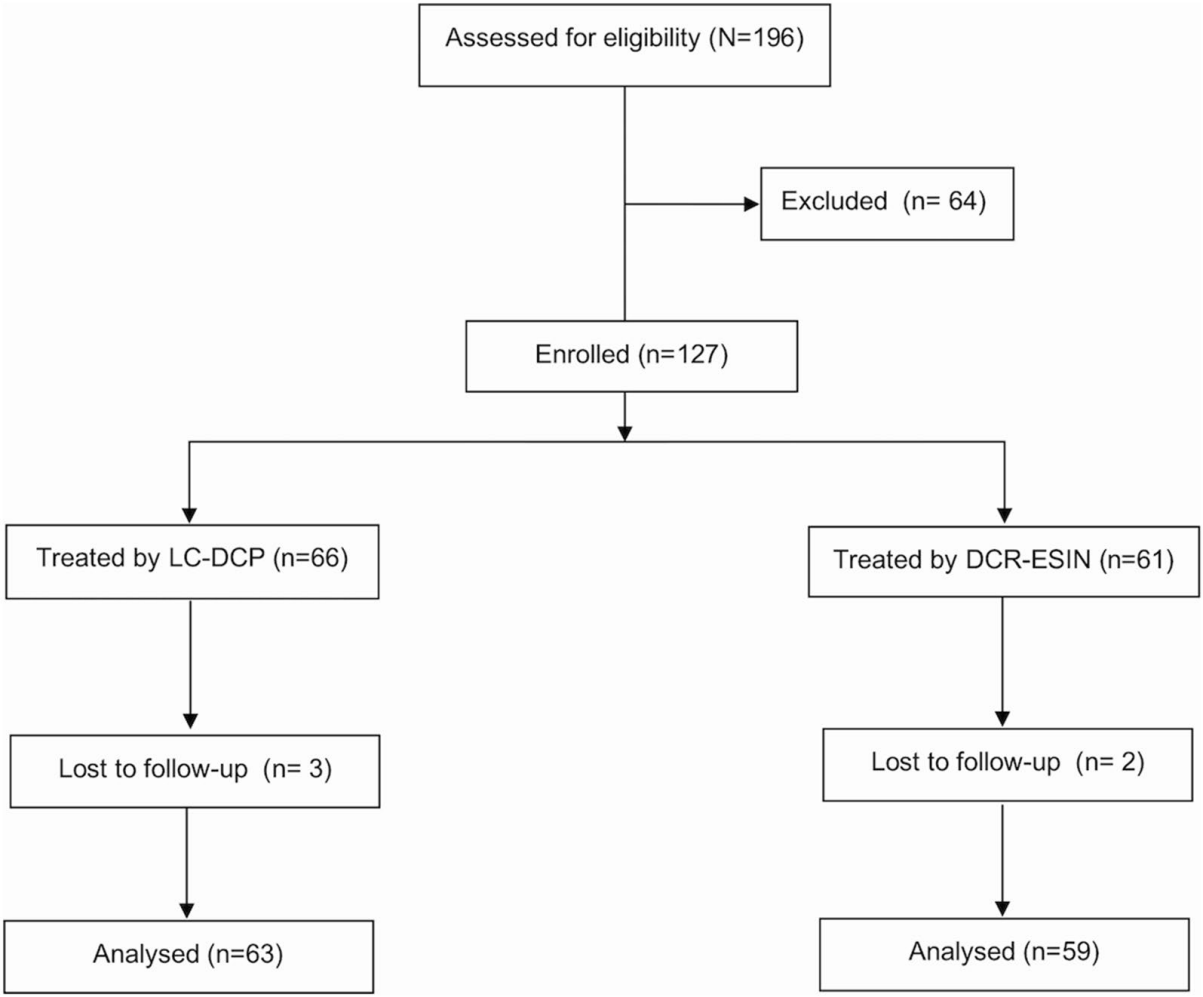
CONSORT flow diagram.* LC-DCP* limited-contact dynamic compression plate,* DCR-ESIN* double-crossed retrograde elastic stable intramedullary nail. Excluded (*n* = 64): revision surgery (*n* = 4), pathologic fracture (*n* = 12), pediatric fracture (*n* = 38), periprosthetic fracture (*n* = 3), intra-articular or metaphyseal extension (*n* = 6)

A total of 196 patients were included in this study. Of those, 69 patients were excluded because they were pediatric, periprosthetic, pathological cases, or lost to follow-up, as seen in the CONSORT diagram (Fig. 2). Analyses were performed on 122 humeral shaft fractures receiving surgical treatment with plates or nails. Plating management was performed in 63 patients, while DCR-ESIN management was performed in 59 patients. There was no difference between the two groups regarding demographic data (Table 1), fracture types, and location (Table 2). Significant differences were detected in the nail group, which had less intraoperative blood loss and shorter operative times (Table 1). The specific outcomes are detailed in Table 3.

**Table 1:** Demographic and perioperative data

	Overall (*n* = 122)	DCR-ESIN (*n* = 59)	LC-DCP (*n* = 63)	*p*-value
Age, mean ± SD, yr	55.34 ± 21.19	55.07 ± 20.28	55.59 ± 22.18	0.893
Gender				0.181
Female	70 (57.4%)	38 (64.4%)	32 (50.8%)	
Male	52 (42.6%)	21 (35.6%)	31 (49.2%)	
Comorbidity				
Diabetes mellitus	25 (20.5%)	11 (18.6%)	14 (22.2%)	0.791
Smoking	18 (14.8%)	8 (13.6%)	10 (15.9%)	0.917
Alcohol	17 (13.9%)	6 (13.6%)	11 (17.5%)	0.368
Obesity	18 (14.8%)	10 (16.9%)	8 (12.7%)	0.685
Psychiatric history	12 (9.8%)	4 (6.8%)	8 (12.7%)	0.428
Operative time, mean ± SD, min	103.8 ± 41.8	76.4 ± 27.98	129.5 ± 36.00	< 0.001
Additional wire fixation	22 (18%)	14 (23.7%)	14 (23.7%)	0.178
Intraoperative blood loss, mean ± SD, ml	155.8 ± 169.02	60.9 ± 61.55	244.8 ± 188.7	< 0.001
Follow-up time, mean ± SD, weeks	148.1 ± 38.51 (67.0–266.0)	150.82 ± 38.51 (98.3–237.1)	145.3 ± 47.95 (67.0–266.0)	0.443

*DCR-ESIN* double-crossed retrograde elastic stable intramedullary nailing, *LC-DCP* limited-contact dynamic compression plate

**Table 2:** Fracture patterns and locations

	Overall (*n* = 122)	DCR-ESIN (*n* = 59)	LC-DCP (*n* = 63)	*p*-value
Fracture patterns				0.664
Simple (12-A)	54 (44.3%)	27 (45.8%)	27 (42.8%)	0.428
Spiral (12-A1)	12 (9.9%)	7 (11.9%)	5 (7.9%)	
Oblique (12-A2)	15 (12.3%)	8 (13.6%)	7 (11.1%)	
Transverse (12-A3)	27 (22.1%)	12 (20.3%)	15 (23.8%)	
Wedge (12-B)	48 (39.3%)	21 (35.6%)	27 (42.9%)	0.860
Spiral wedge (12-B1)	21 (17.3%)	10 (16.9%)	11 (17.5%)	
Bending wedge (12-B2)	25 (20.5%)	10 (16.9%)	15 (23.8%)	
Fragmented wedge (12-B3)	2 (1.5%)	1 (1.8%)	1 (1.6%)	
Complex (12-C)	20 (16.4%)	11 (18.6%)	9 (14.3%)	0.673
Spiral (12-C1)	12 (9.8%)	6 (10.2%)	6 (9.5%)	
Segmental (12-C2)	3 (2.5%)	2 (3.4%)	1 (1.6%)	
Irregular (12-C3)	5 (4.1%)	3 (5.0%)	2 (3.2%)	
Fracture location				0.789
Proximal third	27 (22.1%)	12 (20.3%)	15 (23.8%)	
Middle third	65 (53.3%)	31 (52.5%)	34 (54.0%)	
Distal third	30 (22.1%)	16 (27.1%)	14 (22.2%)	

*DCR-ESIN* double-crossed retrograde elastic stable intramedullary nailing, *LC-DCP* limited-contact dynamic compression plate

**Table 3:** Outcomes in the nail group and plate group

	Overall (*n* = 122)	DCR-ESIN (*n* = 59)	LC-DCP (*n* = 63)	*p*-value
Timely union	116 (95.1%)	59 (100.0%)	57 (90.5%)	0.052
Mean time to union, weeks	13.37 ± 3.19	12.00 ± 2.13	14.8 ± 3.49	< 0.001
Delayed union	3 (2.5%)	0 (0%)	3 (4.8%)	
Mean time to delayed union, weeks	28.62 ± 0.59	–	28.62 ± 0.59	
Nonunion	3 (2.5%)	0 (0%)	3 (4.8%)	
Radiographic angulation				
AP angulation	4.77 ± 3.77 (0.23–21.75)	4.92 ± 4.10 (0.47–21.75)	4.62 ± 3.44 (0.23–15.04)	0.196
Lat. angulation	5.42 ± 4.43 (0.17–25.48)	6.18 ± 5.35 (0.17–25.48)	4.66 ± 3.15 (0.49–13.59)	0.2759
Total complications	17 (12.3%)	3 (5.1%)	14 (22%)	0.006
Radial nerve injury or impingement	3	0	3	0.049
Implant related	4	1	3	0.129
Infection	2	0	2	0.143
Bone healing (delayed union or nonunion)	6 (4.9%)	0 (0%)	6 (9.5%)	0.027
DASH score at 1-year follow-up	8.03 ± 7.50 (0–45)	6.32 ± 3.6 (0–20)	9.63 ± 9.7 (0–45)	0.014

*DCR-ESIN* double-crossed retrograde elastic stable intramedullary nailing, *LC-DCP* limited-contact dynamic compression plate, *AP* anterioposterior,* Lat.* lateral, *DASH score* the disability of the arm, shoulder and hand score

**Fig. 3:**
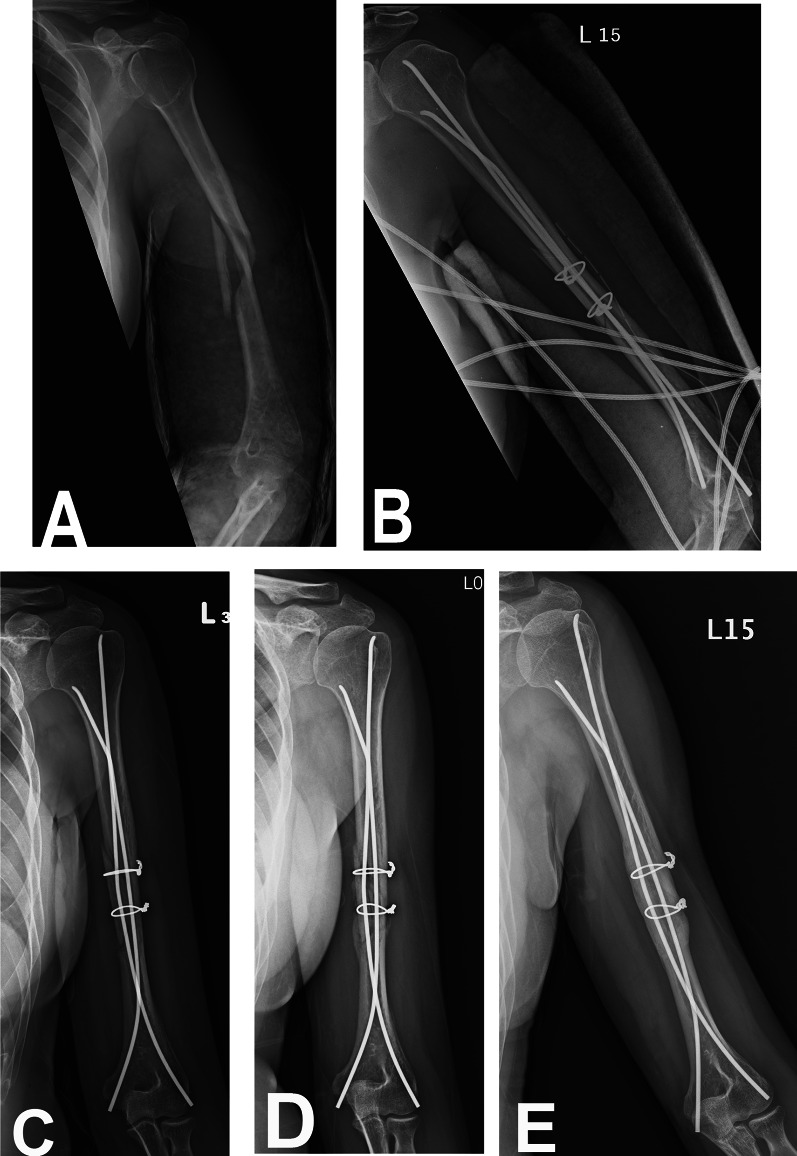
Case demonstration of a 61-year-old female patient: **a** preoperative anteroposterior radiograph of the humerus; **b** postoperative radiograph; **c** postoperative radiograph at 4 weeks’ follow-up; **d** postoperative radiograph at 8 weeks’ follow-up, showing that the bridging callus and fracture line were diminished; **e** postoperative radiograph at 6 months’ follow-up, showing solid union

In terms of primary outcomes, the union rate was 90.5% at a mean of 14.8 weeks in the plating group, while the union rate was 100% at a mean of 12.0 weeks in the DCR-ESIN group. Although there was no significant difference in the union rate (*p* = 0.052), a shorter union time was noted in the DCR-ESIN group (*p* < 0.001). Of the remaining patients, six patients (9.5%) in the plating group did not achieve union. Three patients (4.8%) in the plating group had delayed union at a mean of 28.6 weeks. Three patients (4.8%) in the plating group had nonunions and underwent subsequent autograft revision surgery, with all showing union after the revision surgery. Figure 3A–E shows examples of the nail group, including preoperative, postoperative, and follow-up radiographs.

The rate of overall complications was 5.1% (*n* = 3) and 22% (*n* = 14) in the nail group and plating group, respectively. One case from the DCR-ESIN group experienced nail backout with skin impingement followed by removal of the nails under local anesthesia. There were no complications associated with bone healing, radial nerve injury, or impingement in the nail group. In the plating group, three cases experienced radial nerve injuries or implant impingements. Two of the cases had temporary neuropraxia with recovery after postoperative month 2. The other case had implant impingement with forearm numbness and a positive Tinel’s sign over the posterior aspect of their distal humerus. Neurolysis and removal of the plate were performed after bony union and the symptoms subsided. In addition, two cases had surgical site infections: one was treated with oral antibiotics and the other received local debridement. Three cases had loosening screws that required prolonged sling use, with union achieved in all cases at follow-up.

In terms of secondary outcomes, the average radiographic angulation in the AP view was 4.92° in the nail group and 4.62° in the plating group. The average radiographic angulation in the lateral view was 6.18° in the nail group and 4.66° in the plating group. There was no significant difference in angulation in either the AP or lateral view (Table 3). In addition, the average DASH scores at the 1-year follow-up were 6.32 in the nail group and 9.63 in the plating group (*p* = 0.014).

## Discussion

The present study is the first large cohort study to compare the LC-DCP and DCR-ESIN techniques for fixing diaphyseal fractures of the adult humerus. Although several studies proved that locking plates were superior to LC-DCPs in cadaveric models [10–12], the clinical results showed no difference between locking plates and LC-DCPs [6]. In our study, a major finding was that the DCR-ESIN technique had shorter union times and less complications than the LC-DCP technique. The results indicate that the DCR-ESIN may be an alternative internal fixation for diaphyseal fractures of the adult humerus.

Surgical fixation of diaphyseal fractures generally involves plating or nailing. Kurup et al. [13] conducted an intervention review of five small trials to compare dynamic compression plating vs. locked intramedullary nailing for humeral shaft fractures in adults. No significant difference in fracture union, operating time, blood loss during surgery, or iatrogenic radial nerve injury between the two fixation methods was found. However, there was a statistically significant increase in shoulder impingement following nailing when compared with the LC-DCP technique. Use of the DCR-ESIN technique can theoretically avoid shoulder impingement or cuff violations. In addition, explorations were minimally invasive. In our study, the DCR-ESIN group had superior functional outcomes and less complications than the LC-DCP group, as discussed in the following paragraphs.

Chen et al. [14] and Chiu et al. [15] reported that the Ender nail, a flexible intramedullary nail, had superior outcomes with regard to blood loss, operative times, and union times. However, those two studies mixed the two entry methods of the nail group. Moreover, the retrograde methods used in the two aforementioned studies were based on methods from DeLong et al. [16] and were different from our methods. We believe that the inevitable backout and loss of rotational stability may happen. Using our DCR-ESIN methods, a three-point fixation may reduce the inevitable backouts and strengthen the rotational stability [7].

Discussions involving the union and nonunion of humeral diaphyseal fractures have led to controversies in recent years [9]. Maresca et al. [17] concluded that there were three main factors in humeral shaft nonunion: fracture type, grade of open fracture, and type of osteosynthesis. In our study, the LC-DCP group and the DCR-ESIN group showed similar results in union rates. However, the DCR-ESIN group had shorter union times than the LC-DCP group. These results indicate that the evacuation of hematoma plus periosteal stripping in the plating group may cause prolonged union times or increase the probability of nonunion or delayed union.

Infections, prolonged union times, and radial nerve palsy are general concerns about the LC-DCP technique [18]. Conventional techniques involve an extensive surgical approach for the open reduction of fractures [19]. In our study, three cases in the plating group required oral antibiotics or local debridement for surgical site infections, while there were no cases of infection in the DCR-ESIN group. In addition, radial nerve palsy, including temporary and permanent radial nerve injury, was also found in the LC-DCP group, with no radial nerve injury in the DCR-ESIN group. Due to the extensive surgical approach, the radial nerve would be retracted for a prolonged period of time, potentially resulting in ischemia due to manipulation or small vessel destruction or potential damage caused by implants or drilling [20–22].

With the DCR-ESIN technique, the two different entry points may reduce cortical cracking, and the nail, pre-bent into a ‘C’ curve, may provide three-point fixation. Two double-crossed nails can provide rotational stability. Additional wire was suggested when encountering long-spiral or large-wedge fragments, as it provides additional stability and more bone contact. This retrograde method can prevent the shoulder stiffness generated in the antegrade method.

There are some limitations of our study. This study had a retrospective design, which may have introduced selection bias and, therefore, been more susceptible to data loss and confounding than a prospective study. Additionally, subgroups were too small to be independently identified and analyzed. In our study, we did not intend to debate operative versus nonoperative treatment of diaphyseal fracture of the adult humerus. The choice between nonoperative and operative treatment of this fracture should be made on an individual basis. In addition, a postoperative CT scan, which was not included in the National Health Insurance, was not obtained as a means to evaluate rotation. Even though around one-quarter of the patients in the DCR-ESIN group needed additional wiring with a minimally invasive technique, the remaining three-quarters of the patients benefited from closed reduction with DCR-ESIN instrumentation. However, the most suitable technique for a particular fracture pattern cannot be determined based on this study. Further research is therefore warranted for different fracture patterns. In addition, we suggest that a prospective randomized controlled trial could be performed to compare these two methods.

## Conclusion

This study has demonstrated that the DCR-ESIN technique for diaphyseal humeral fractures can provide shorter operative times, less intra-operative blood loss, and shorter union times than the conventional LC-DCP technique. The DCR-ESIN technique may be an alternative method for treating diaphyseal humeral fractures in adults.

## Data Availability

The information needed to access the data used in the study is included within this article.
